# Different Fluorophore Labeling Strategies and Designs Affect Millisecond Kinetics of DNA Hairpins

**DOI:** 10.3390/molecules190913735

**Published:** 2014-09-03

**Authors:** Andreas Hartmann, Georg Krainer, Michael Schlierf

**Affiliations:** 1 B CUBE, Center for Molecular Bioengineering, Technische Universität Dresden, Dresden 01307, Germany; E-Mails: hartmann@bcube-dresden.de (A.H.); krainer@bcube-dresden.de (G.K.);; 2 Molecular Biophysics, University of Kaiserslautern, Kaiserslautern 67663, Germany

**Keywords:** DNA hairpin, labeling strategy, single-molecule FRET, hairpin design

## Abstract

Changes in molecular conformations are one of the major driving forces of complex biological processes. Many studies based on single-molecule techniques have shed light on conformational dynamics and contributed to a better understanding of living matter. In particular, single-molecule FRET experiments have revealed unprecedented information at various time scales varying from milliseconds to seconds. The choice and the attachment of fluorophores is a pivotal requirement for single-molecule FRET experiments. One particularly well-studied millisecond conformational change is the opening and closing of DNA hairpin structures. In this study, we addressed the influence of base- and terminal-labeled fluorophores as well as the fluorophore DNA interactions on the extracted kinetic information of the DNA hairpin. Gibbs free energies varied from ∆*G*^0^ = −3.6 kJ/mol to ∆*G*^0^ = −0.2 kJ/mol for the identical DNA hairpin modifying only the labeling scheme and design of the DNA sample. In general, the base-labeled DNA hairpin is significantly destabilized compared to the terminal-labeled DNA hairpin and fluorophore DNA interactions additionally stabilize the closed state of the DNA hairpin. Careful controls and variations of fluorophore attachment chemistry are essential for a mostly undisturbed measurement of the underlying energy landscape of biomolecules.

## 1. Introduction

The analysis of biomolecular conformational dynamics is of fundamental importance for a better understanding of complex biological processes. Single-molecule techniques have the power to directly resolve distributions and heterogeneities of individual biomolecular complexes underlying the ensemble average and follow unsynchronized dynamics that might remain masked when large numbers of molecules are probed simultaneously [1,2]. In particular, single-molecule Förster Resonance Energy Transfer (smFRET) has become a powerful and popular tool to explore and quantify conformational dynamics of nucleic acids [3,4,5,6,7,8,9,10,11,12,13,14,15,16,17,18,19,20,21,22] and proteins [23,24,25,26,27,28,29,30,31,32,33,34,35]. smFRET experiments quantify the energy transfer efficiency between a single donor and acceptor fluorophore pair. The non-radiative energy transfer strongly depends on the inter-fluorophore distance and has a characteristic length (Förster radius) on the order of a few nanometers, thus, matching the length scales of most biomolecules. smFRET can be used as a molecular ruler for biological systems and is a complementary technique to methodologies, such as X-ray crystallography, NMR or electron microscopy, supplementing structural information with dynamic content. Nowadays, two conceptually different approaches are frequently used for smFRET experiments [36]: surface immobilized studies and confocal spectroscopy with freely-diffusing molecules. Surface immobilized FRET studies based on total-internal-reflection fluorescence (TIRF) microscopy allow the observation of slow conformational dynamics (tens of milliseconds to minutes) of individual molecules, while confocal spectroscopy combined with time-correlated single-photon counting (TCSPC) on both, freely-diffusing and surface immobilized molecules, enables the observation of distinct populations with fast interconversion dynamics (microseconds to milliseconds) [37].

**Figure 1:**
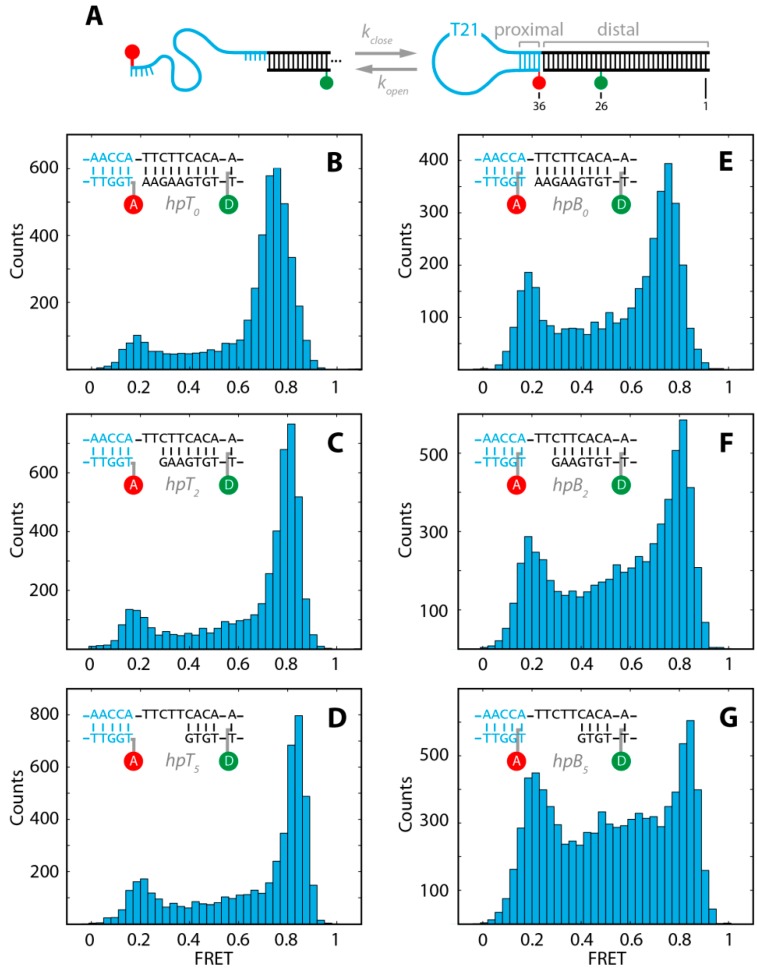
(**A**) Schematic for the conformational fluctuation of the stem-loop DNA hairpin between closed and open states with the characteristic opening rate and closing rate, *k*_open_, *k*_close_, respectively .The basic DNA hairpin consists of a T21 loop and a 5 bp proximal stem (5'-AACCA-3'), followed by a 25–30 bp long distal stem. All constructs were labeled at position 26 with ATTO532 and at position 36 with ATTO647N; (**B**–**G**) FRET efficiency histograms of the six different DNA hairpins after removal of donor-only and acceptor-only populations and photobleaching events (see text).

Among the studies on dynamic biomolecules in living systems at the single-molecule level, investigations of the conformational changes of DNA and RNA hairpins have become one of the main topics. Hairpins display a relatively simple structural element that consists of inverse repeats of single-stranded DNA connected by a non-complementary loop region. Depending on their stem length, hairpins constantly undergo changes between folded (closed) and unfolded (open) conformations with different kinetic rates. Nucleic-acid hairpins are highly conserved genetic elements involved in DNA recombination, gene transcription and DNA replication [38]. RNA hairpins are, for example, involved in regulating protein translation by forming temperature-responsive hairpin structures [39,40]. Moreover, hairpins are also used in nanotechnological applications as biosensors and reporters (e.g., as molecular beacons or anti-sense dugs) in diagnostics and drug development [41,42]. Thus, a fundamental understanding of hairpin structures and their underlying energy landscape is essential. Opening and closing kinetics of hairpins have been studied in detail previously by means of smFRET and complementary fluorescence techniques reported to depend strongly on sequence, stem length, salt concentration and temperature [9,10,11,12,13,14,15,16,17,43,44,45,46,47,48,49,50,51]. In these studies two fundamentally different hairpin designs were used. The first design is a minimal model consisting of a single-stranded DNA with a central loop and complementary sequences at the ends, resembling molecular beacons. Fluorophores were attached in this design to both, the 3'- and 5'-end (e.g., [9,10]). The second design (e.g., [11,12,13,15,16] uses an additional single-stranded DNA to separate the donor and acceptor fluorophore to avoid dye-dye stacking that could lead to quenching and additional blinking [52]. This design creates a proximal stem that contains the complementary hairpin sequence and a long distal stem that contains the donor or acceptor fluorophore (Figure 1A).

The attachment of fluorescent probes, preferentially organic fluorophores, is a pivotal step to report on conformational changes between the open and closed state in hairpins by monitoring differences in the FRET efficiency. Among the wealth of different labeling approaches available for site-specific modification of DNA and RNA nucleotides the most commonly used strategies comprise either conjugation of the fluorophores to the 5'- or 3'-end of the nucleotide (terminal labeling) or at internal positions of nucleobase derivatives (base labeling). For terminal labeling on the 5'- or 3'-ends, fluorophores are covalently linked to the terminal phosphate group. This conjugation is accomplished mostly either via phosphoramitide-based chemistry or via an amine reaction to an amino-functionalized aliphatic linker attached to the terminal phosphate group. In base-labeling, nucleotide bases of pyrimidines (e.g., thymidines) modified with an aliphatic amino linker attached to the C5 ring are conjugated with amine-reactive functionalized fluorophores.

In respect to the different labeling strategies, extreme care has to be taken not to perturb residues or regions that might be involved in the formation of interactions and thereby severely destabilize the folded structure. This in turn may interfere with the actual conformational dynamics of the biomolecule to be observed and, thus, change its characteristics of the underlying energy landscape [14,53]. Moreover, fluorophore properties can be altered depended on the local microenvironment of the biomolecule [54,55,56,57,58,59].

To date, little is known about the influence of distinct labeling strategies and the distal stem on the dynamic equilibrium between the folded and unfolded conformations. Changes of the physicochemical properties of DNA or RNA introduced by different positioning of the labels and distal stems, could potentially affect the thermodynamic stability of hybridization and, consequently, the underlying energy landscape. A solid interpretation of these changes, however, requires detailed knowledge of the influence of the fluorophores on the conformational transitions.

In this study, we addressed the influence of the labeling strategies and the design of the distal stem on the millisecond kinetics of a DNA hairpin using diffusion-based pulsed interleaved excitation smFRET burst analysis with multiparameter fluorescence detection [60,61,62,63]. Millisecond kinetics of DNA hairpin formation have been extracted using recently developed analysis methods based on the approximation of the FRET efficiency distribution by a sum of three-Gaussian functions [64,65]. We found that base-labeled hairpins are destabilized compared to the terminal-labeled hairpins and, thus, can lead to a misinterpretation of the state and rate constants. Additionally, distal stem designs stabilized the closed hairpin conformation significantly.

## 2. Results and Discussion

### 2.1. DNA Hairpins with Millisecond Kinetics

**Figure 2:**
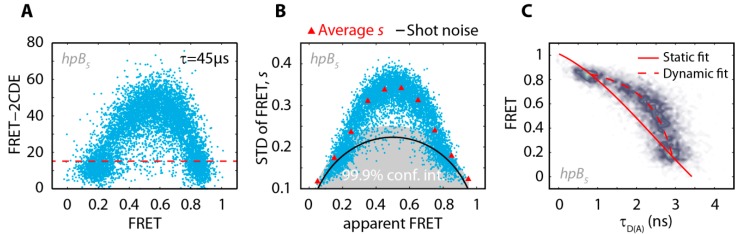
(**A**) Scatter plot of FRET-2CDE *vs.* FRET for the DNA hairpin construct hpB_5_. The FRET-2CDE value (τ = 45 μs, kernel time constant) was calculated for every burst. Well-defined closed and opened conformations were selected by the analysis of bursts with a FRET-2CDE value below 15 (threshold indicated by red dashed line); (**B**) An equivalent method is the Burst variance analysis (BVA). Static, well-defined open or closed species can be found at the shot noise line (black line) within the confidence interval of 99.9% (grey); (**C**) FRET *vs.* τ_D(A)_ for the DNA hairpin construct hpB_5_. The displaced stream between the two FRET states shows that the mid-population in Figure 1G originates from fast interconverting molecules.

In order to investigate the influence of different labeling strategies and distal stem designs for a DNA hairpin with millisecond kinetics, we used a hairpin construct (Figure 1A) with a T21 loop region and a 5 bp proximal stem, containing a GC content of 40%. The distal stem length was varied between 25 bp and 30 bp to introduce a gap of zero, two or five nucleotides between the proximal and distal stem (insets in Figure 1B–G). The 5 bp sequence of the proximal stem and the length of the loop were chosen such that the opening and closing kinetics are on the millisecond time-regime [12,13,15,16,17]. Conformational changes were probed with the FRET pair ATTO532 and ATTO647N as donor and acceptor fluorophores, respectively. The donor fluorophore was attached to the distal stem via base labeling (position 26). The acceptor was conjugated to the proximal stem at the 5'-end (position 36) either via base (hpB) or terminal labeling (hpT) (Figure 1A). In the closed (folded) conformation the distance of donor and acceptor result in a high efficiency of energy transfer (high FRET), in the open (unfolded) conformation the increased distance between donor and acceptor lead to a low efficiency of energy transfer (low FRET). The two differently labeled proximal stems in combination with the three different distal stem designs result in six constructs (hpB_0_, hpB_2_, hpB_5_, hpT_0_, hpT_2_, hpT_5_; insets Figure 1B–G and Section 3) that were measured in single-molecule burst measurements at a constant temperature (*T* = 21.4 ± 0.1 °C) and identical buffer conditions. Pulsed-interleaved excitation combined with multiparameter fluorescence detection (MFD) allowed us to separate FRET populations from donor-only, acceptor-only and photobleaching events by the use of several filter algorithms (see Section 3) [62]. Figure 1B–G show the FRET histograms after removal of the above mentioned donor-only, acceptor-only and photobleaching events. As expected, two well-defined peaks representing the open and closed state of the DNA hairpin were measured. FRET events between the folded and unfolded peak do not represent a third static species, but can be identified using methods like a two-channel kernel-based density distribution estimator (FRET-2CDE) for photon distribution analysis, or burst variance analysis (BVA), or a FRET *vs.* donor lifetime (τ_D(A)_) plot to be molecules changing their conformation during the diffusion through the confocal volume (see Figure 2 and Section 3).

The kinetic bursts were removed using a FRET-2CDE cut-off of 15 (Figure 2A) and the remaining static open and closed populations were fitted with Gaussians to determine the open and closed FRET efficiencies (Table 1).

The FRET efficiency histograms including the kinetic bursts (as shown in Figure 1) were further analyzed with a three-Gaussian (3G) fit [65]. This model assumes two well-defined states (two-state model), which interconvert on a time scale that is comparable to the diffusion time (< *t*_D_ > = 2 ms):

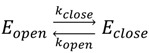
(1)


**Table 1:** The average FRET values with the related standard deviations of the open and closed state for the six different DNA hairpin constructs obtained from *N*_mol_ molecules.

Substrate	*E*_open_ ± σ_open_	*E*_close_ ± σ_close_	*N*_mol_
hpT_0_	0.17 ± 0.05	0.77 ± 0.05	1622
hpT_2_	0.16 ± 0.04	0.82 ± 0.04	1440
hpT_5_	0.19 ± 0.05	0.85 ± 0.05	1589
hpB_0_	0.17 ± 0.04	0.77 ± 0.04	1058
hpB_2_	0.18 ± 0.05	0.83 ± 0.05	1267
hpB_5_	0.19 ± 0.05	0.86 ± 0.05	1713

By fitting the FRET histogram with the three-Gaussian distributions that are coupled to each other (see Section 3), opening and closing rates on the millisecond time scale (Table 2) can be extracted. The FRET peak amplitudes reflect the ratio of the opening and closing rate *k*_open_/*k*_close_. The FRET efficiency peak between the closed and open state originates from molecules changing their conformation during the diffusion through the confocal volume and, thus, the more area is contained in the center population, the more frequent conformational changes occur.

**Table 2:** The opening and closing rates of the DNA hairpin constructs, the related changes in Gibbs free energy (∆*G*^0^)and the transition state free energy barriers (∆*G*^‡^).

Substrate	*k*_close_ (ms^−1^)	*k*_open_ (ms^−1^)	∆*G*^0^ (kJ/mol)	∆*G*^‡^_close_ (kJ/mol)	∆*G*^‡^_open_ (kJ/mol)
hpT_0_	0.86 ± 0.05	0.20 ± 0.01	−3.57 ± 0.26	19.98 ± 0.13	23.55 ± 0.11
hpT_2_	0.92 ± 0.05	0.29 ± 0.01	−2.83 ± 0.22	19.81 ± 0.13	22.64 ± 0.12
hpT_5_	0.91 ± 0.02	0.35 ± 0.02	−2.34 ± 0.19	19.84 ± 0.06	22.18 ± 0.12
hpB_0_	0.66 ± 0.04	0.41 ± 0.03	−1.17 ± 0.33	20.63 ± 0.15	21.79 ± 0.18
hpB_2_	0.87 ± 0.02	0.55 ± 0.01	−1.12 ± 0.10	19.95 ± 0.06	21.07 ± 0.05
hpB_5_	0.90 ± 0.01	0.84 ± 0.01	−0.17 ± 0.06	19.87 ± 0.03	20.04 ± 0.04

Surprisingly, even though the proximal stem of the hairpin was kept identical in the different samples, we observed significant changes in the FRET populations and kinetic rates of the hairpin depending on the labeling strategy and on the gap between the proximal and distal stem (Figure 1, Table 1 and Table 2).

### 2.2. The Base-Labeled Hairpin is More Dynamic than the Terminal-Labeled Hairpin

In a first set of analysis, we compared the three terminal-labeled constructs (Figure 1B–D) with the three base-labeled constructs (Figure 1E–G). We found that the FRET efficiencies of the corresponding terminal- and base-labeled hairpins are identical within our resolution for the open and closed populations, respectively (Table 1). This indicates that regardless of the labeling strategy the open and closed states are identical. A striking difference between both labeling strategies can be found in the ratio of the peak amplitudes and the inter-peak FRET efficiency populations. The terminal-labeled constructs (Figure 1B–D) exhibited less dynamics and were predominantly closed while the base-labeled constructs (Figure 1E–G) were more dynamic and resided more often in the open state.

As expected from the shape of the FRET efficiency distribution, the 3G analysis revealed that the opening rate of all base-labeled constructs (hpB) are approximately twice as high compared to all terminal-labeled constructs (hpT) (Table 2). This indicates that either hpT is stabilized by the fluorophore attachment at the terminal end or hpB is destabilized by the C6-linker attached fluorophore to the base.

In contrast, the closing rates are identical among the corresponding hairpins except for *k*_close_ of the construct hpB_0_, which is reduced by 25% compared to the other closing rates. The closing rate is related to the probability of an end-to-end collision of the DNA hairpin and, thus, is mainly dominated by the loop length and electrostatic repulsion [43,44]. In all our constructs the loop length and salt concentration was identical, therefore, we conclude that in the construct hpB_0_, the end-to-end collision probability is reduced with this labeling strategy.

Overall, the apparent Gibbs free energy differences between the open and closed state are significantly different between the two different labeling strategies with a ∆∆*G*^0^(hpT_n_-hpB_n_) ≈ 2 kJ/mol (Table 2). Thus, the base-labeled hairpin is generally strongly destabilized compared to the terminal-labeled hairpin.

The closing and opening rates further allowed us to calculate transition state free energies *(*∆*G***^‡^**_open_, Δ*G***^‡^**_close_, Table 2) using Kramers’ theory with a previously extrapolated pre-exponential factor *k*_0_ = 3 × 10^6^ s^−1^ [66] (see Section 3). The free energy barrier heights for closing, Δ*G***^‡^**_close_, are almost identical for the six constructs whereas the barrier heights for opening of the hairpins, Δ*G***^‡^**_open_, are varying and decreasing with increasing gap size and are generally lower in the terminal labeled constructs than in the base labeled constructs. Since the thermodynamic stability, ∆*G*^0^, is the difference between Δ*G***^‡^**_open_ and Δ*G***^‡^**_close_, this implies that the stability among the different labeling strategies and hairpin designs is mainly influenced by the barrier height of the opening process. We compared our experimentally extrapolated barrier heights to a theoretical prediction of the free energy difference for opening of the hairpin (Δ*G***^‡^**_open_) as established previously [16,17]. To this end, the free energy required for melting a five base pair duplex DNA with our sequence using the nearest-neighbor method [67] (MFOLD, [68,69]) was calculated. The calculation yielded a value of 20 kJ/mol for the hybridization free energy matching within error the experimentally determined opening barriers. In a previous study, Nir and coworkers [17] determined barrier heights for opening of a similar hairpin construct that was base labeled and had a gap size of two nucleotides between the proximal stem and the distal stem. They reported experimentally derived barrier heights using Kramers’ theory about 0.5–0.7 kJ/mol higher than the melting energy of the corresponding duplex using MFOLD. Remarkably, the barrier height of opening of our corresponding construct hpB_2_ is in good agreement with their offset, however, our construct hpB_5_ with increased gap size yielded a barrier height that was exactly matching to the calculated MFOLD value of duplex formation. In general, based on the observed differences in the barrier height, the pre-exponential factor can be adjusted to match MFOLD predictions for either of the hairpin constructs as done previously [17], that would yield *k*_0_ = 2.0 × 10^6^ s^−1^ for hpB_2_ and *k*_0_ = 3.1 × 10^6^ s^−1^ for hpB_5_. Our data, however, indicates that a small system like this DNA hairpin can be disturbed by the labeling position of the fluorophore leading to potential destabilizations during base labeling or stabilizations due to DNA fluorophore interactions (see Section 2.3). Extrapolations of pre-exponential factors from the experimental data can thus be severely hampered by the sample design and an independent determination of the pre-exponential factor is crucial for further interpretations.

### 2.3. The Acceptor Fluorophore on the Hairpin Interacts with the Double-Stranded Distal Stem

**Figure 3:**
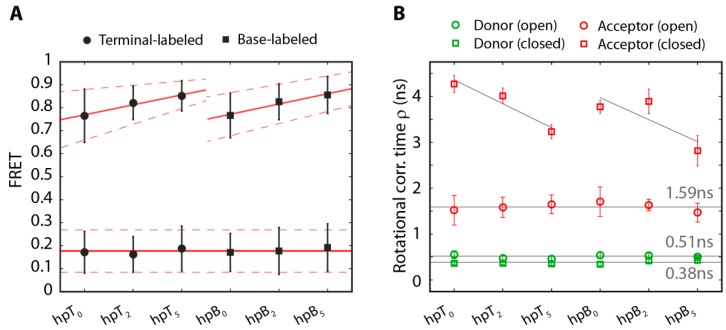
(**A**) FRET efficiencies of the low FRET (open) and high FRET (closed) population, with two sigma deviations, for the 5'-modified terminal- and the base-labeled DNA hairpin constructs. The data were extracted from bursts with FRET-2CDE smaller than 15; (**B**) Related steady state rotational correlation times, obtained from steady state anisotropy fits by the Perrin equation (lines are a guide to the eye).

In a next set of analysis, we determined the influence of the distal stem on the proximal stem by increasing the distance between both stems from zero to two to five nucleotides for both labeling strategies. The FRET efficiencies of the open state did not change significantly (Figure 3A and Table 1). Interestingly, increasing the gap between both stems, led to an increase in the FRET efficiencies for the closed state (Figure 3A and Table 1). The FRET increase is not unexpected due to the reduced persistence length of single-stranded DNA compared to double-stranded DNA and thus a reduced time-averaged end-to-end distance in both constructs.

**Figure 4:**
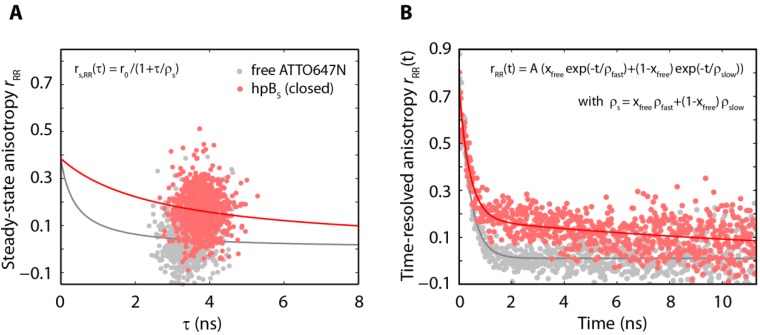
Global fitting of the steady state (**A**) and time-resolved anisotropy (**B**) for hpB_5_ in the closed conformation (red) reveals two rotational components. The fast decay originates from the freely rotating acceptor dye and the slow decay from the sticking of the acceptor dye to the hairpin. The freely rotating acceptor molecule (gray) shows a steady state rotational correlation time corresponding to the fast component in the hairpin and only a single exponential decay in the time-resolved anisotropy.

At the same time, for both, terminal-and base labeled hairpins the opening rate *k*_open_ increases with the gap size between the proximal and the distal stem (Table 2). The terminal-labeled and the base-labeled constructs with a gap of five nucleotides showed an increase in the opening rate of about 75% and 100%, respectively, compared to those with a zero nucleotide gap. The increase of the opening rate was in both labeling schemes anti-correlated with the steady state anisotropy rotational correlation time, ρ_s,closed_, of the acceptor in the closed state (Table 2 and Figure 3B). Moreover, a detailed global fitting analysis of the steady state anisotropy together with the time-resolved anisotropy exhibited for all constructs a fast and a slow component of the acceptor closed state rotational correlation times, ρ_fast_ and ρ_slow_, respectively (exemplarily shown for hpB_5_ in Figure 4A, red). The fast decay phase with rotational correlation times of approximately 0.3–0.5 ns (e.g., ρ_fast_(hpB_5_) = 0.33 ± 0.16 ns) indicates a freely rotating fluorophore in agreement with the rotational correlation time ρ_s_ = 0.39 ns of freely diffusing acceptor molecules (Figure 4A, gray) and values from literature of DNA-bound acceptor molecules [70]. The slow decay time on the other hand indicates a restrained fluorophore with rotational correlation times of 13–19 ns (e.g., ρ_slow_(hpB_5_) = 15 ± 6 ns). We quantified the relative amplitude, *x*_free_, of the fast rotational correlation time of the acceptor in the closed conformation for all constructs (Table 3). For both labeling strategies, these fractions were increasing with larger gaps between the proximal and distal stem for both, terminal- and base-labeled hairpin constructs. A larger gap size from zero to five freed the acceptor by approximately 10%. This increase lowers the averaged steady state rotational correlation time significantly by 30% due to the weight of the slow component of the time-resolved rotational correlation time. The constrained acceptor rotation in the closed hairpin can originate from several factors. A molecular reasoning of this constrain is rather complicated and probably includes several properties of the fluorophore, like hydrophobicity, charge, size, and rigidity of the linker and dye itself. Possible scenarios for the DNA hairpin can be stacking on the terminal basepairs or the interaction with the major groove of the distal stem (limited by the C6 linker length) [56,57,71,72,73]. This additional interaction can stabilize the closed hairpin structure and thus reduce the opening rate in all constructs.

**Table 3:** Rotational correlation times, relative amplitudes of fast rotational correlation times and the steady state rotational correlation times in the closed hairpin conformation of the acceptor.

Substrate	ρ_*fast*_ (ns)	ρ_*slow*_ (ns)	x_*free*_	ρ_*s,closed*_ (ns)
hpT_0_	0.47 ± 0.11	15 ± 3	0.74 ± 0.07	4.27 ± 0.19
hpT_2_	0.37 ± 0.10	14 ± 3	0.74 ± 0.06	4.01 ± 0.17
hpT_5_	0.51 ± 0.08	13 ± 3	0.79 ± 0.06	3.23 ± 0.15
hpB_0_	0.34 ± 0.09	14 ± 4	0.76 ± 0.08	3.77 ± 0.14
hpB_2_	0.41 ± 0.12	19 ± 8	0.81 ± 0.08	3.89 ± 0.27
hpB_5_	0.33 ± 0.16	15 ± 6	0.84 ± 0.08	2.82 ± 0.33

The closing rates of the constructs, on the other hand, were mainly unaffected by the gap size between the proximal and the distal stem, except of the previously mentioned closing rate of hpB_0_. Here, increasing the gap size from zero nucleotides to two nucleotides (hpB_0_ -> hpB_2_) increased the closing rate by 25% (Table 2). Increasing the gap from two to five nucleotides (hpB_2_ -> hpB_5_) did not further increase the closing rate constant within our experimental resolution. Therefore, a minimal gap of two nucleotides seems to be sufficient to reduce the steric hindrance in the base-labeled construct.

Interestingly, the nearly constant closing rates correlate well with the steady state anisotropy rotational correlation times ρ_s,open_ of the acceptor for the open hairpins that were independent of the labeling strategy and constant at a value of around 1.6 ns. Time-resolved anisotropy of the open state revealed again a fast and a slow component with nearly constant ratios and a fast rotational correlation time around 0.4 ns (e.g., ρ_fast_(hpB_5_) = 0.37 ± 0.16 ns) and a slow component of around 4 ns (e.g., ρ_slow_(hpB_5_) = 4 ± 1 ns).

The microenvironment of the donor fluorophore was unaffected by hairpin opening and closing. All constructs yielded a donor lifetime of τ_D(0)_ = (3.43 ± 0.03) ns, and the steady state anisotropy rotational correlation time for the open and closed conformation ρ_s,open_ = 0.51 ns and ρ_s,closed_ = 0.38 ns, respectively, was constant.

## 3. Experimental Section

### 3.1. DNA Hairpin Design and Labeling Strategies

**Figure 5:**
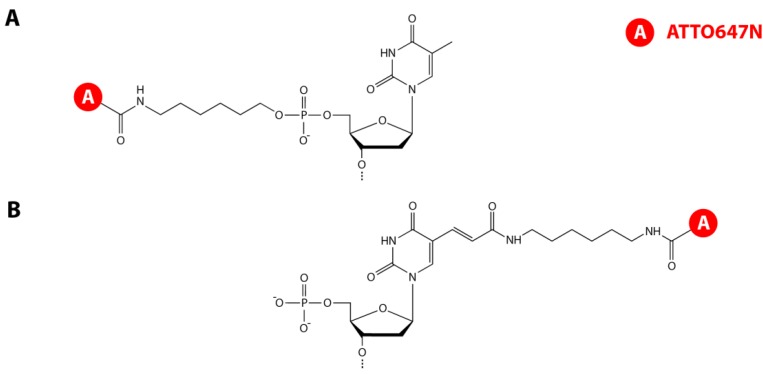
Chemical drawings of the acceptor labeling strategies. (**A**) In terminal labeling the acceptor is coupled to the amino-C6-modified 5'-phosphate-thymidine; (**B**) In base labeling the acceptor is attached to an amino-C6-modified thymine.

Fluorescently labeled and HPLC-purified DNA single strands were obtained from IBA (Göttingen, Germany) modified with the corresponding *N*-hydroxysuccinimidyl ester (NHS) donor and acceptor fluorophore derivatives of ATTO532 and ATTO647N (ATTO-TEC, Siegen, Germany). The acceptor was conjugated with NHS-chemistry to the thymidine on the 5'-end of the top strands either via terminal or base labeling (see Figure 5).

In terminal labeling, the acceptor was covalently linked to the amino-C6-modified 5'-phosphate whereas in base labeling the acceptor was attached to an amino-C6-modified thymidine (dTC6). The donor was labeled at position 26 of the bottom strands via NHS-chemistry to the dTC6 base. Top strand hpT_0,2,5_: 5'-ATTO647N-TGGTT-(T)_21_-AACCATTCTTCACAAACCAGTCCAAACTATCAA AACTTA-3'; Top strand hpB_0,2,5_: 5'-dTC6-NH-(ATTO647N)-GGTT-(T)_21_AACCATTCTTCACAAA CCAGTCCAAACTATCACAAACTTA-3'; Bottom strand hpT_0_/hpB_0_: 5'-TAAGTTTGTGATAGTTT GGACTGGTdTC6-NH-(ATTO532)-TGTGAAGAA-3'; Bottom strand hpT_2_/ hpB_2_: 5'-TAAGTTTGT GATAGTTTGGACTGGTdTC6-NH-(ATTO532)-TGTGAAG-3'; Bottom strand hpT_5_/hpB_5_: 5'-TA AGTTTGTGATAGTTTGGACTGGT dTC6-NH-(ATTO532)-TGTG-3'.

The DNA hairpin constructs were annealed at a concentration of 5 µM in 10 mM Tris-HCl pH 8.0 (Roth, Karlsruhe, Germany), 50 mM NaCl (Roth) and 1 mM EDTA (AMRESCO, Solon, OH, USA) by heating to 90 °C and cooling to room temperature in 1 K/min steps.

### 3.2. smFRET Measurements and Analysis Procedures

Freely diffusing single molecule measurements were performed in a 20 mM Tris-HCl (Roth) pH 8 buffer, containing 250 µM EDTA (AMRESCO), 175 mM NaCl (Roth). To minimize blinking of the fluorophores an aged saturated Trolox-solution was added to the buffer. The Trolox-solution (6-hydroxy-2,5,7,8-teramethylchroman-2-carboxylic acid, Sigma-Aldrich, St. Louis, MI, USA) was prepared freshly and aged over night at 4 °C [74]. Multiple molecule detection was avoided by a very diluted (5 pM) hairpin solution and samples were measured in a Methoxy-PEG (5 kDa, Rapp Polymere, Tübingen, Germany) passivated home-built flow-chamber [75]. During the measurements the temperature of the sample was controlled by objective cooling to avoid environmental effects on the melting behaviour of the DNA. The temperature of the slide was monitored with a 230 µm-sized temperature probe (Physitemp Instruments, Clifton, NJ, USA) attached to the top of the chamber. The temperature remained stable with ∆*T* < 0.2 K for all measurements. The individual values of the mean temperatures, for each measurement, are shown in Table 4.

**Table 4:** Mean temperature of the single molecule measurements.

Substrate	*T* (°C)
hpT_0_	21.34 ± 0.09
hpT_2_	21.34 ± 0.07
hpT_5_	21.41 ± 0.09
hpB_0_	21.36 ± 0.15
hpB_2_	21.34 ± 0.07
hpB_5_	21.38 ± 0.14

**Figure 6:**
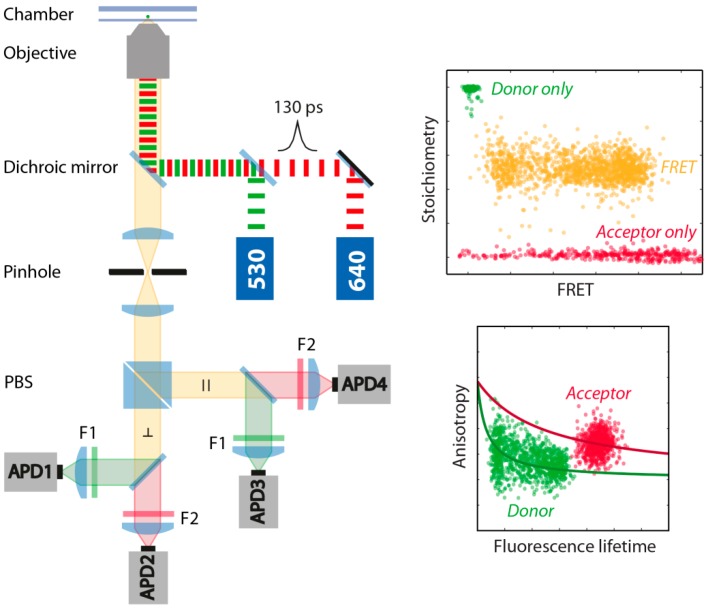
Schematic illustration of the home-built confocal TCSPC microscope. Donor and acceptor fluorophores were excited at 530 nm and 640 nm with pulsed-interleaved excitation (PIE) with a total repetition rate of 50 MHz to remove donor only and acceptor only molecules from the analysis. Donor and acceptor anisotropy and fluorescence lifetime are measured simultaneously to obtain information about the microenvironment and rotational freedom of the fluorophores. A stoichiometry *vs.* FRET plot (*S*-*E*-plot) allows the identification of FRET molecules (orange), donor-only molecules (green) and acceptor-only molecules (red). PBS: polarizing beam splitter; APD: avalanche photo diode; F1/F2: bandpass filter.

FRET experiments were performed in a home-built confocal microscope combined with Time-Correlated Single Photon Counting (TCSPC) (Figure 6). For the donor and acceptor excitation we used two pulsed laser sources with wavelengths of 530 nm and 640 nm (LDH-P-FA-530L and LDH-D-C-640, Picoquant, Berlin, Germany). The two lasers were driven in the pulsed interleaved excitation (PIE) mode (PDL828 “Sepia II”, Picoquant) with a total repetition rate of 50 MHz and a delay time of 20 ns and a laser power of 178 µW for the green laser and 156 µW for the red laser measured before the objective. The dye emission was collected 70 µm in solution by a 60x water objective (CFI Plan Apo 60x Water, Nikon, Tokyo, Japan) with a numerical aperture of 1.2. For the separation of illumination and detection pathway, we used a dual-edge dichroic mirror (zt532/640rpc, Chroma, Bellows Falls, VT, USA) and for the separation of the donor and acceptor emission, after a polarizing beam splitter (PBS), a single-edge dichroic mirror (FF650-Di01, Semrock, New York, NY, USA). Emitted photons were bandpass filtered (F1: FF01-582/75, Semrock; F2: ET700/75M, Chroma), detected by four single-photon avalanche diodes (τ–SPADs, Picoquant) and registered by four independent channels of a TCSPC module (HydraHarp 400, Picoquant).

From the acquired photon stream only bursts with an inter-photon time smaller than 25 µs and a minimum total number of 200 photons were selected. To remove molecules affected by photobleaching, an asymmetric burst filter was applied, which discards all bursts with an absolute difference of the burst-averaged macroscopic photon arrival time measured in the acceptor channel after acceptor excitation and the burst-averaged macroscopic photon arrival time measured in the donor and acceptor channel after donor excitation bigger than 50 µs [62]. For the remaining molecules the corrected stoichiometry and FRET values, *S* and *E*, respectively, were derived from burst intensities as follows:

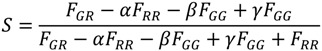
(2)

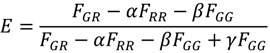
(3)


Here, *F*_GG_ and *F*_GR_ are the background subtracted fluorescence intensities in the donor and the acceptor channel, respectively, after donor excitation and F_RR_ in the acceptor channel after acceptor excitation. The detection correction factor γ and the correction factors for direct excitation, α, and crosstalk, β, were extracted from the gained *S*-*E* plot (α = 0.038, β = 0.014 and γ = 0.7) [62]. Only bursts with a stoichiometry between 0.3 and 0.7 were used for further analysis.

Population averaged steady state rotational correlation times, time-resolved rotational correlation times and fluorescence lifetimes were determined as described [62,63]. Both, the steady state anisotropy and the time-resolved anisotropy, were fitted globally using the Perrin equation and a double-exponential decay, respectively (see insets Figure 4).

We used a two-channel kernel-based density distribution estimator (2CDE) for photon distribution analysis, to separate static molecules from those that undergo multiple transitions during diffusion through the detection spot [15]. After calculating the FRET-2CDE value (τ = 45 µs, kernel time constant) for every molecule, static FRET bursts could be easily separated from dynamic bursts by applying a threshold of 15 (Figure 2A). Additionally, we used burst variance analysis (BVA) [13] as an alternative method to resolve dynamic bursts from static bursts (Figure 2B). To identify FRET fluctuations within a burst the standard deviation of the mean FRET efficiency is calculated for individual molecules with a five photon binning of the burst trace. Molecules with an underlying FRET distribution in their burst trace show an increased standard deviation *s*, (see Figure 2B) whereas static FRET species can be found at 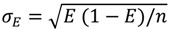 with *n* = 5, the number of photons.

**Figure 7:**
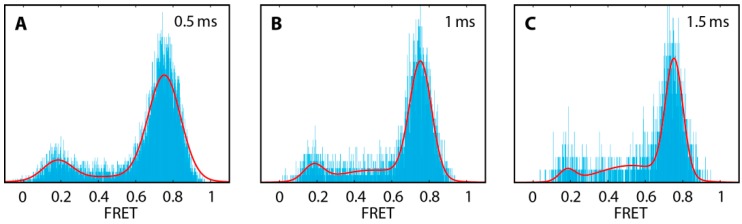
Global fit of the DNA hairpin construct hpT_0_ using the three-Gaussian approximation [65]. The three FRET efficiency histograms were obtained from different bin times: 0.5 ms (**A**), 1 ms (**B**) and 1.5 ms (**C**). A small FRET bin size of 0.001 was chosen to minimize bin size artefacts.

Kinetic rates were extracted from FRET histograms using the three-Gaussian approximation for a two-state system described by Gopich and Szabo [65]. The three-Gaussian function is defined by the FRET efficiencies of the open and closed state *E*_open_ and *E*_close_, the opening and the closing rate *k*_open_ and *k*_close_, the bin time *T* and the average of the inverse of the total number of photons 〈*N*^−1^〉. The average of the inverse of the total photons and the bin time were derived from the measurement, whereas *E*_open_, *E*_close_, *k*_open_ and *k*_close_ were freely varied. The FRET states and the conversion rates were obtained from a global fit to a set of three FRET efficiency histograms generated from the same measurement but using different bin times within a burst: 0.5 ms, 1 ms and 1.5 ms (see Figure 7). Bin size artefacts and errors of least-squares fits were minimized by a FRET bin size of 0.001.

The Gibbs free energy difference was derived from:

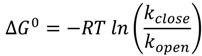
(4)
with *R* being the universal gas constant, *T* the absolute temperature and *k*_open_ and *k*_close_ are the opening and closing rates, respectively (see Table 2).

The height of the Gibbs free energy barriers for opening and closing of the hairpins in a one-dimensional free energy profile was calculated using Kramers’ transition-state theory (see Table 2):

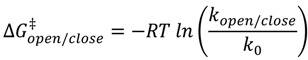
(5)
where *k*_open_ and *k*_close_ are the observed opening and closing rates, respectively, Δ*G***^‡^**_open_ and Δ*G***^‡^**_close_ are the free energy barrier heights with respect to the free energies of the closed and the open state, respectively, and the pre-exponential factor *k*_0_, reflecting the transition rate in the absence of a free energy barrier. In our calculations, we used the previously extrapolated pre-exponential factor *k*_0_ = 3 × 10^6^ s^−1^ [66].

## 4. Conclusions

In this study, we showed that the opening and closing rates of a millisecond folding hairpin, monitored by smFRET, are strongly influenced by the labeling scheme of the proximal stem and the distance to the adjacent distal stem. While the microenvironment of the donor, attached to the distal stem, is identical within our experimental error, the acceptor fluorophore was a major determinant in affecting the folding kinetics of the hairpin. The kinetic analysis revealed that base-labeled hairpins are destabilized compared to the terminal-labeled hairpins. This weakening could originate from a disturbed adenine-thymine bond by the fluorophore attached to the thymine base. On the other hand, we observed a stabilizing effect of the closed conformation, which decreased with increasing gap size between the proximal and distal stem. Analysis of the time-resolved anisotropy of the acceptor dye suggests that this originates from a dye-DNA interaction. Increasing the gap distance between both stems led to an increase in the fraction of freely rotating acceptor. Furthermore, we found that the base-labeling in the hpB_0_ construct reduced the closing kinetics of the hairpin significantly, most likely due to steric hindrance. It is important to note, that in all our studies the inherent hairpin structure of the loop length and the five annealing bases was unchanged, however, the deduced apparent free energy between the closed and open state changed significantly from ∆*G*^0^(hpT_0_) = −3.6 kJ/mol to ∆*G*^0^(hpB_5_) = −0.2 kJ/mol. Our data using multi-parameter fluorescence detection of fluorophore anisotropy indicate that the constructs hpT_5_ and hpB_5_ reflect the least disturbed systems. With the assumption that an undisturbed system is the most stable system, the hpT_5_ construct would be the experimental candidate of choice. On the other hand, in light of the match of the theoretical barrier height for opening and the experimentally extrapolated barrier height the hpB_5_ construct would be the preferred hairpin.

Previous studies showed that the choice of the FRET pair severely affects the information deduced from single-molecule FRET experiments [14]. We found that an additional important factor for the design of single-molecule FRET experiments is also the labeling scheme. Especially studies that analyze biomolecular kinetics on the millisecond timescale, e.g., DNA or RNA hairpin formation, protein folding, conformational changes in enzymes, are prone to be significantly affected by destabilizing and stabilizing effects of fluorophores attached to the biomolecule. Thus, it is crucial to vary FRET pairs and the attachment chemistry to minimize the effect of the labeling chemistry. Taking these precautions into account for the design of experiments, single-molecule FRET remains one of the most powerful techniques to study structure-function relationships and underlying energy landscapes of biomolecules.
